# The T-Blep: A Soft Optical Sensor for Stiffness and Contact Force Measurement

**DOI:** 10.3390/mi15020233

**Published:** 2024-02-01

**Authors:** Federico Bernabei, Matteo Lo Preti, Lucia Beccai

**Affiliations:** 1 Soft BioRobotics Perception, Istituto Italiano di Tecnologia, 16163 Genova, Italy; matteo.lopreti@iit.it; 2 The BioRobotics Institute, Scuola Superiore Sant’Anna, 56127 Pisa, Italy

**Keywords:** soft sensor, stiffness detection, force detection, multifunctional sensor, optical sensor

## Abstract

This paper presents the Tactile Blep (T-Blep), an optical soft sensor that can measure the stiffness and force of different materials. The sensor consists of an inflatable membrane with an optical elements inside. The T-Blep can switch between stiffness detection and force detection modes, by changing the pattern followed by internal pressure of the membrane. Simulations reveal that a 1 mm-thick membrane enables differentiation of extra-soft, soft, and rigid targets. Furthermore, the sensitivity and FSO of the force estimation can be adjusted by varying the internal pressure. Force detection experiments exhibit a sixfold increase in detectable force range as internal pressure varies from 10 kPa to 40 kPa, with a force peak of 5.43 N and sensitivity up to 331 mV/N. A piecewise force reconstruction method provides accurate results even in challenging conditions ( $R^{2}>0.994$ ). Stiffness detection experiments reveal distinguishable patterns of pressure and voltage during indentation, resulting in a classification accuracy of 97%.

## 1. Introduction

Touch sense helps us to instinctively gather crucial information from our surroundings, enabling us to explore and navigate in unfamiliar environments. In the medical field, palpation is used in breast examinations [1,2,3], prostate checks [1], and initial tumor identification [2]. Similarly, in agriculture, a gentle squeeze of a fruit, detailed by Erkan et al. [4], offers vital tactile feedback for assessing ripeness, highlighting touch’s pivotal role in exploring and manipulating our surroundings.

The importance of touch takes center stage also in robotics. Integrating tactile feedback into robotic systems can significantly reduce grasping forces during operations [5] and provide feedback in vision occluded scenario, e.g., surgical operation [6]. This reduction translates into energy savings and could also contribute to prevent the target from damages due to an excess of grasping force.

Traditionally, haptic feedback relies on applied force to ensure that robotic systems exert sufficient force on known and well-defined targets. However, there are still open challenges in scenarios where the target is unknown and deformable. In such cases, predefining the grasping force can lead to overestimation, risking object damage, or underestimation, resulting in grip loss. To address these issues, a preliminary non-destructive investigation of the stiffness of the target emerges as a crucial strategy. This approach facilitates determining the correct force range required for a successful grasp, thereby leveraging the adaptability and precision of robotic manipulations [7]. The focus on stiffness detection becomes a pivotal factor in optimizing the efficacy of robotic interactions with unpredictable and deformable targets.

Stiffness sensing entails quantifying the resistance of a material or fabric to deformation under applied force. Conventionally, this is accomplished through the use of a rigid indenter with known properties, allowing the reconstruction of the elastic modulus via the Hertz-Force indentation relation [8,9] or application-specific relations [10,11]. Despite their common use in portable solutions, these approaches often encounter challenges in adaptation and integration into robotic grippers due to miniaturization issues and the necessity to guarantee a load in a direction normal to the target surface. An alternative approach, which allows for miniaturization, involves employing two or more sensing elements with distinct elastic constants to activate a capacitive sensor [3,12,13,14,15]. Through a comparative analysis of the capacitive sensor’s response due to a different displacement in correspondence with these elements, the estimation of the target material’s stiffness becomes feasible. These solutions, being based on capacitive transduction mechanisms, typically exhibit characteristics of linearity and temperature invariance [16,17,18]. Nonetheless, they are susceptible to electromagnetic interference and commonly necessitate complex measurement units [16,17,18]. Furthermore, their capacity to solely measure displacement requires the use of further sensing elements to provide the system with contact force information, making integration challenging where space is limited, such as a robotic finger.

Versatile solutions that can simultaneously assess stiffness and force, by using the inverse magnetostrictive effect, have been recently introduced [19,20]. In these cases, the authors leveraged the interaction between Galfenol cantilevers and permanent magnets to detect force and displacement. The reconstruction of the stiffness of the targeted materials was then effectively achieved through the application of Hooke’s law. While the use of magnetic transduction makes these solutions suitable to measure (with high-resolution) force in the range 0 N to 5 N and the stiffness of the target. The use of magnetic elements introduces biases by an external magnetic field or by the interaction with ferromagnetic elements in the environment [16,17,18], reducing the range of possible applications.

In this study, we present a tunable optical soft sensor designed for the dual detection of stiffness and force. Unlike conventional methodologies employing capacitive and magnetic transduction mechanisms, our approach integrates optical elements, ensuring high resolution while robustly responding in the presence of electromagnetic fields [16,17,18].

The T-Blep comprises an inflatable membrane housing an optical sensor element. Its functionality as either a force or stiffness sensor is contingent upon the pressurization mode. In dynamic pressure conditions, the sensor operates as a stiffness detector, employing a k-nearest neighbors algorithm (KNN) to categorize the target into three distinct softness levels: extra-soft, soft, and rigid. Conversely, under static pressure, it seamlessly transitions into a force detection mode. The T-Blep introduces a versatile and adaptive solution developed with a view to future integration on a robotic grasping system.

## 2. Materials and Methods

### 2.1. Structure and Principle of the Sensor

**Figure 1:**
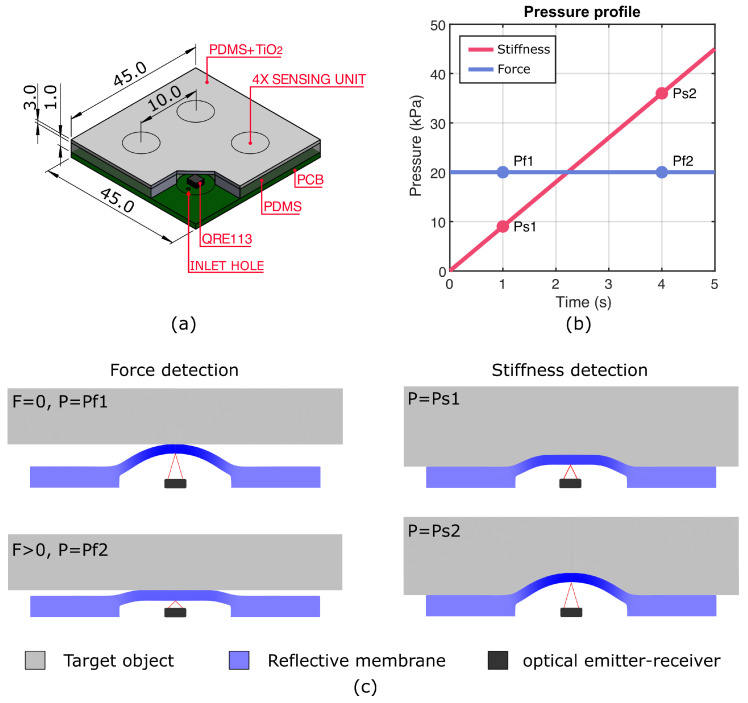
Working Principle and T-Blep details. (**a**) Schematic representation of the fully assembled T-Blep array, where silicone multi-material layer and PCB are attached through silicone adhesive. The four bleps are indicated by circles spaced by 10 mm. All the indicated dimensions are in mm; (**b**) Pressure profiles adopted during force and stiffness detection tasks. While the ramp for stiffness detection remains constant, the pressure value for force detection can be adjusted based on the targeted force range; (**c**) Working principle in stiffness detection (ramp pressure pattern) and force detection (constant pressure).

The structure and dimension of the sensor used in the experimental activity are represented in Figure 1a, where an array of four T-Bleps is presented. The array of detection units and the relative electronics were chosen to fabricate multiple sensors at once and to take into account variability in each unit’s response. Moreover, having four distinct contact points for stiffness detection allows to recognize small differences in the dataset due to the natural local inhomogeneity of the material. Hence, the gathered data resulted more representative of real-world scenarios. Each sensor array comprises four optoelectronic sensing elements equally distributed at a distance of 10 mm between four chambers on the surface and oriented to measure force and displacements along the direction normal to the surface. As shown in the section of the single sensing element visible in Figure 1a, the T-Blep consists of three main layers: (i) the flexible printed circuit board (PCB), (ii) the bottom non-reflective layer, and (iii) the top reflective layer. While the PCB is a flexible layer, the top and bottom layers are made of soft silicone. This comes from the need to create an inflatable membrane that deforms to relatively low pressure. Hence, materials that fulfill mechanical requirements such as significant elongation at break, low elastic modulus, and high stretchability are required.

The operating principle of the sensor is based on the QRE113 miniature reflective object sensor (Semiconductor Components Industries LLC, Scottsdale, AZ, USA), as reported in Figure 1c. Schematically, it can be represented by an integrated circuit comprising a photodiode and a phototransistor. QREE113 uses the change in collector current to measure the distance between the sensing element and the reflective surface of the target by emitting and receiving light in the infrared range ( ∼940 nm). The initial distance between the optoelectronic elements and the top layer was chosen according to the characteristic response of QRE113, to allow force and stiffness detection depending on the actuation pattern. As shown in Figure 1b,c, force sensing is obtained by applying a constant and positive pressure within the chambers, such that the response to contact results smoother. Differently, when the applied pressure follows a ramp pattern from 0 k Pa to 45 k Pa , the trend of the indentation depth allows the system to recognize the stiffness of the target.

### 2.2. Fabrication Process and Materials

The fabrication process and materials used were chosen to minimize fabrication steps while still meeting the mechanical and optical requirements for proper sensor operation. Both were built of Polydimethylsiloxane (PDMS) (Sylgard 184, Dow Inc., Midland, MI, USA) at a 10:1 mix ratio to ensure proper bonding between the bottom and top silicone layers. However, to fabricate an adequately reflective top layer, 4.3 wt% of Titanium dioxide particles (Ti $O_{2}$ ) were added to the initial formulation of PDMS, following the procedure reported in [21]. In both cases, the formulation was mixed for 30 s using a Thinky ARE-250 mixing and degassing machine (THINKY USA, INC, Laguna Hills, CA, USA), followed by an additional degassing step of 5 min using the S-26P-NL vacuum degassing system (Easy Composites EU BV, Rijen, The Netherlands).

**Figure 2:**
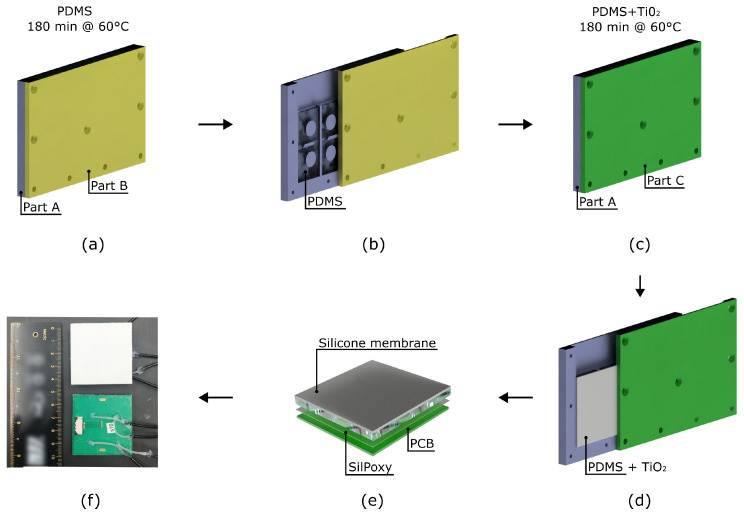
T-Blep fabrication process. (**a**,**b**) Manufacturing steps to fabricate the silicone layer made of PDMS; (**c**,**d**) Manufacturing steps to fabricate the to complete the silicone membrane with the reflective layer; (**e**) The membrane and the Printed Circuit Board (PCB) are joined using Sil-Poxy adhesive; (**f**) Finalized sensor configuration with pneumatic silicone tubes attached to the inlet holes, securely sealed with SilPoxy adhesive.

Based on casting techniques, the fabrication followed the steps presented in Figure 2. The three-piece mold reported in Figure 2a–d was designed using SolidWorks (Dassault Systems SolidWorks Corporation, Waltham, MA, USA) and 3D printed by fused deposition modeling (FDM) with an Ultimaker S3 (Ultimaker B.V., Geldermalsen, The Netherlands) using an acrylonitrile butadiene styrene filament (ABS, Ultimaker B.V., Geldermalsen, The Netherlands), suitable for oven curing. Once parts A and B of the mold were assembled, PDMS was poured inside the system. The mold was placed in the degassing chamber for 5 min to remove any trapped air pocket, then cured at 60 ${\;}^{\circ }C$ for 1.5 h . Once the first step was completed, part B of the mold was replaced with part C, and the process described in the initial step was repeated, but this time using the PDMS+Ti $O_{2}$ formulation. To conclude, as reported in Figure 2e,f, the resulting two-layer silicone part was removed from the mold, aligned with the sensing elements on the PCB, and attached to the PCB layer using a one-component silicone adhesive Sil-Poxy™ (Smooth-On, Inc., Macungie, PA, USA), taking special care to tightly seal the four chambers and the tube used to provide pressure during actuation.

### 2.3. Simulation and Experimental Activity

To adequately characterize the sensor under the two operating conditions, finite element analysis (FEA) simulations and experimental tests were performed.

#### 2.3.1. FEA Analysis

**Figure 3:**
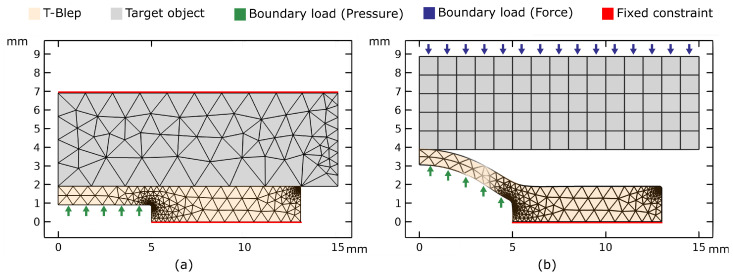
2D axisymmetric model employed in the FEA study with a membrane thickness of 1 mm. (**a**) Mesh configuration utilized for stiffness simulation. (**b**) Mesh design utilized for force analysis, considering the T-Blep pressurized to a specified pressure.

The FEA study was conducted in COMSOL Multiphysics^®^ 5.6 (COMSOL Inc., Stockholm, Sweden), assuming the 2D representation of the sensing element of the T-Blep depicted in Figure 3. Since the objective of the simulation was to verify the existence of a correlation between the displacement of the top layer and both target stiffness and force applied to the contact point (by the T-Blep on the target object), a simplified model not including the optical component of the sensor was investigated. In addition, given the symmetry of the sensing unit, a 2D axisymmetric model was used in the simulation. The top and bottom layers, made of the same silicone material (PDMS), were represented as a unique body for both studies.

For the stiffness analysis, the sensor and target were assumed to be in contact before inflation. A fixed constrain was applied to the bottom boundary of the T-Blep and the top boundary of the target, while a distributed pressure (P) ranging from 0 k Pa to 45 k Pa was applied to the T-Blep membrane as shown in Figure 3a. While the latter was modeled as a hyperelastic material, assuming a Neo-Hookean model, the target substrate was modeled as an elastic material. For simplicity, for both bodies a free-tetrahedral mesh was assumed.

For force analysis, the two bodies were assumed to be in contact. However, as shown in Figure 3b, the T-Blep was previously deformed by a fixed internal pressure (*P*). Moreover, the fixed constraint was applied only to the bottom boundary of the T-Blep, while the target body was assumed free to move along the vertical direction due to a normal load (*F*). Unlike the stiffness study, only the T-Blep was modeled as hyperelastic material, while the target was assumed to be an elastic, undeformable solid body. In both cases, a parametric study was conducted. While the dimensions of the model are visible in Figure 3, in Table 1, the main parameters used in the two studies are resumed.

#### 2.3.2. Experimental Protocol

Three tests were performed to demonstrate the multi-functionality of the sensor, and the resulting data were post-processed using MATLAB R2023 (The MathWorks, Inc., Natick, MA, USA).

**Figure A1:**
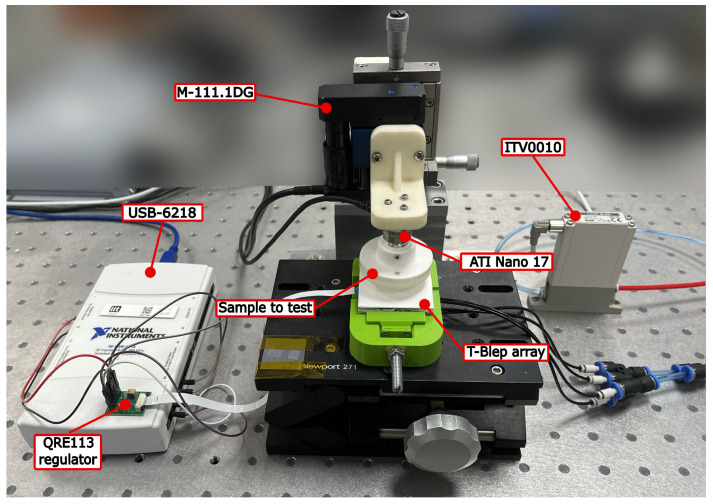
Setup configuration used for the stiffness and force analysis experimental activities.

All the tests were conducted using a custom platform, reported in Figure A1, and repeated on three different samples of the sensor array. A micrometric servo-controlled translation stage M-111.1DG (Physik Instrumente, Karlsruhe, Germany), interfaced with a triaxial load cell ATI Nano 17 (ATI Industrial Automation, Inc., Apex, NC, USA), was used to control the distance between the sensor and the target and recording the force applied. At the same time, a flow regulator ITV0010 (SMC corporation, Tokyo, Japan) was used to set the pressure inside the T-Blep to values ranging from 0 k Pa to 45 k Pa . Two DAQ systems, USB-6218 (National Instruments, Austin, TX, USA), were employed to control the platform, regulate the pressure, and acquire the data with a sampling rate of 50 Hz . At the same time, a custom PCB board was used to regulate the QRE113 light intensity and phototransistor gain.

**Figure 4:**
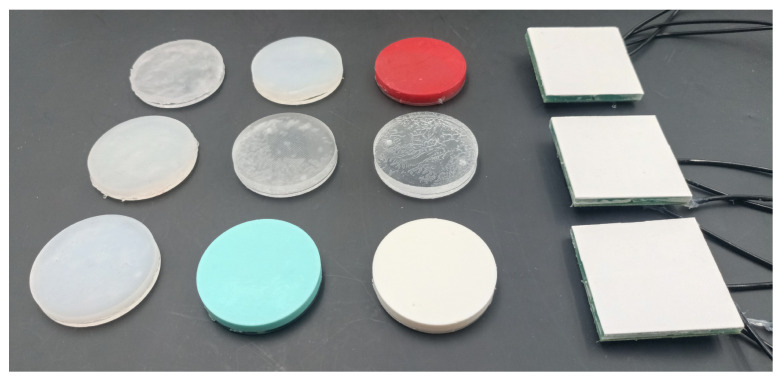
Samples and specimens used in the experimental activity. Arranged in columns from left to right: Extra-Soft specimens, Soft specimens, Rigid specimens, and T-Blep Arrays.

To characterize the stiffness detection, a set of nine specimens was used to generate the dataset to train the classifier to recognize extra-soft, soft, and rigid targets, as show in Figure 4. Each of the specimens used was fabricated having a diameter of 50 m m and a thickness of 5 m m . The material used were Ecoflex Gel™ (Smooth-On, Inc., Macungie, PA, USA), Ecoflex 00-30™ (Smooth-On, Inc., PA, USA), DragonSkin 10™ (Smooth-On, Inc., PA, USA), DragonSkin 30™ (Smooth-On, Inc., PA, USA), Sylgard 186 (Dow Chemical Company, Midland, MI, USA), SmoothSil 960 (Smooth-On, Inc., PA, USA), TPU 95A ((Ultimaker B.V., The Netherlands), ABS (Ultimaker B.V., The Netherlands), and Plexiglass. Each material sample was selected based on its Young’s modulus, as detailed in Table 2. Even though the Young’s modulus of a material with unknown geometry cannot reliably serve as an indicator of stiffness, in the present protocol such an inference is permissible since the geometry is predetermined and equal among samples.

Once the sensor resulted in contact with the desired specimen, 20 inflation cycles from 0 k Pa to 45 k Pa were performed, and the response was recorded. The results were digitally filtered and elaborated through a classifier trained with the K-nearest neighbor algorithm.

A different approach was employed to characterize force detection. Maintaining the pressure inside the T-Blep constant, a flat rigid indenter was used to systematically indent the four sensing elements until contact occurred between the indenter surface and the non-inflated area of the sensor. The experiment was repeated at 10 kPa, 20 kPa, 30 kPa and 40 k Pa . Additionally, 20 indentation cycles were performed for each pressure level to ensure a comprehensive assessment.

#### 2.3.3. Processing Tactile Reading

**Figure 5:**
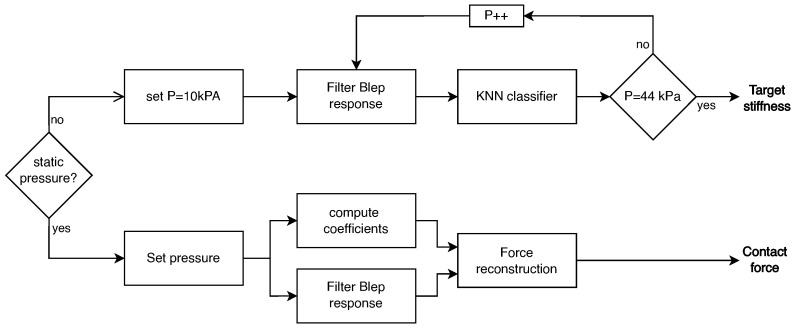
Schematic representation of the methodology for stiffness and force detection. The sensor operates as a stiffness detector during dynamic pressure increase and as a contact force detector under static pressure. Force reconstruction employs a polynomial approach with coefficients computed online, while stiffness detection utilizes a KNN classifier.

The scheme in Figure 5 shows the two approaches used for stiffness and force detection. In both cases, the signal was pre-processed by a digital low-pass filter. In the case of stiffness detection, a dataset was created to train a KNN classifier. The input to the classifier comprises two features, i.e., the current pressure imposed on the regulator and the voltage read by the T-Blep. For each specimen, 1017 points were obtained by concatenating multiple cycles such that the dataset was balanced. Multiple subsets of the dataset were created by limiting the pressure range imposed on the T-Blep to optimize the acquisition procedure. A categorical vector was associated with each row of the dataset with categories extra soft, soft, and rigid. The hyperparameters of the classifier summarized in Table 3 were chosen with a grid search by maximizing the accuracy. Further analysis of the result was implemented by training a KNN with sample hyperparameters using the name of the probe material as output, as reported in Appendix C.

Differently, in the case of force detection, the signal was initially normalized using the equation:

(1) $$\chi =\frac{X-min(X)}{max(X)-min(X)}$$

where:

(2) $$\;X=e^{\left(x_{0}-x\right)}$$

corresponds to the exponential difference between the response with no load and the current one. Finally, the results were elaborated through the system of equations:

(3) $$\begin{array}{c} F\left(\chi \right)=\left\{\begin{array}{cc} H\left(x_{n}-x_{c}\right)\;\cdot \;\left[H\left(x_{c}-\chi \right)\;\cdot \;f_{1}\left(\chi \right)+H\left(\chi -x_{c}\right)\;\cdot \;f_{2}\left(\chi \right)\right]+ & \mathrm{if}\;\chi \leq x_{c}\\ H\left(x_{c}-x_{n}\right)\;\cdot \;\left[H\left(x_{n}-\chi \right)\;\cdot \;C\;\cdot \;f_{1}\left(\chi \right)+H\left(\chi -x_{n}\right)\;\cdot \;f_{2}\left(\chi \right)\right]\\ f_{2}\left(\chi \right) & \mathrm{if}\;\chi >x_{c} \end{array}\right. \end{array}$$

where $f_{1}\left(\chi \right)$ , $f_{2}\left(\chi \right)$ , and *C* are polynomials whose coefficients are functions of the pressure (P) imposed to the T-Blep; H(i) is the Heaviside step function, while $x_{c}$ and $x_{n}$ define the interval of that maximizes the R-square of the force reconstruction function F(χ) for the prescribed *P*.

While (3) allows a direct estimation of force from the normalized signal, the sensitivity and Full Scale Output (FSO) can be adjusted online since the coefficients and parameters used for both normalization and force estimation only depend on the pressure imposed on the regulator within the range 10 k Pa to 40 k Pa . Consequently, this allows for fine-tuning the sensor’s stiffness, thus expanding the range of detectable forces. A comprehensive elucidation of this methodology is provided in the supplementary information reported in Appendix B.

## 3. Results and Discussion

### 3.1. FEM Analysis

**Figure 6:**
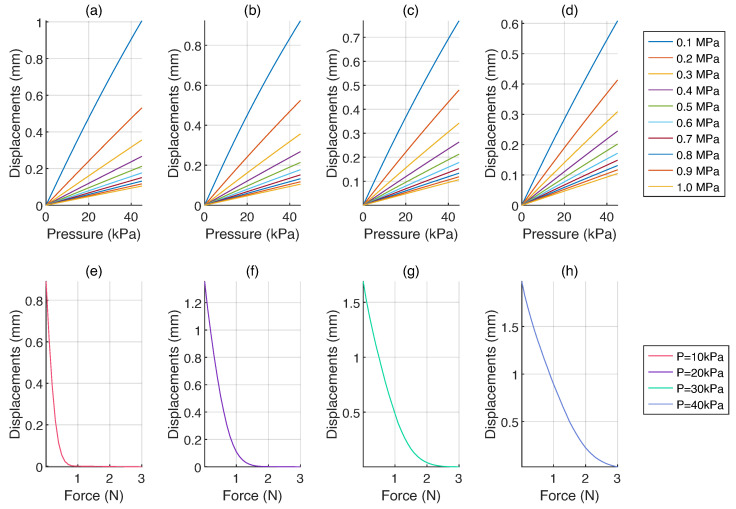
Results of the FEM analysis. (**a**–**d**) Displacement variations in response to changing membrane thickness and target stiffness under increasing pressures in the range 0 kPa to 45 kPa. The configurations are presented as follows: (**a**) Membrane thickness: 0.5 mm; (**b**) Membrane thickness: 1.0 mm; (**c**) Membrane thickness: 1.5 mm; and (**d**) Membrane thickness: 2.0 mm. (**e**–**h**) Displacement variations as a response to force in the range 0 N to 3 N when the T-Blep pressure results fixed at: (**e**) 10 kPa; (**f**) 20 kPa; (**g**) 30 kPa; and (**h**) 40 kPa.

Figure 6 validate in an FEA study the hypothesis of using the same sensor for measuring stiffness and force under different actuation conditions.

The graphs in Figure 6a–d show that variations in the target stiffness correspond to different indentation depths. Regardless of the membrane thickness, stiffer materials exhibit flatter curves, with displacements never exceeding 0.2 mm, even under the case of the maximum pressure (40 kPa). Conversely, softer materials, depending on the membrane thickness, permit more substantial indentations, reaching almost 1 mm for a thickness of 0.5 mm, as reported in Figure 6a.

While a greater indentation depth improves differentiation between soft and hard materials, it is advisable to limit the depth to prevent inducing plastic deformation in weak targets, particularly when the material is unidentified. On the other hand, small deformations may be better suited for brittle materials, but they can also produce false positives in stiffness differentiation. Figure 6d shows that this is especially true for stiffer materials, where most of the tested configurations correspond to indentation between 0.1 mm to 0.2 mm.

After analyzing the results of the simulations, it was determined that a membrane thickness of 1 mm would be appropriate. This choice produces displacement patterns that accurately reflect the material’s stiffness while also avoiding excessive indentation depths in the target subject.

The graphs presented in Figure 6e–h describe the outcomes of force simulation, considering a membrane thickness of 1 mm. In each scenario, the displacement represents the distance from the base of the T-Blep; returning its maximum value at the idle state when no load is applied to the sensor. As expected, the rising internal pressure within each T-Blep induces an increase in the detectable maximum force. This outcome arises from a combination of factors intrinsically linked to the pressure, encompassing the stiffness of individual elements and the initial height of the domes.

Given the extreme cases of pressure at 10 kPa and 40 kPa, the initial dome height goes from 0.91 mm to 1.97 mm and the structure gets more rigid. Consequently, a displacement of 0.5 mm generates a force of 0.15 N at 10 kPa, up to nearly 1.5 N when the imposed pressure is 40 kPa. The same happens assuming intermediate pressure values at 20 kPa and 30 kPa, where the same displacement generates forces equal to 0.52 N and 0.98 N, respectively.

The results of both simulations confirmed how the same sensor can be used as a force sensor or to recognize the stiffness of the target, imposing a static or dynamic pressure, respectively. In the particular case of static pressure, it has been demonstrated that increasing pressure corresponds to greater ranges of measurable force.

### 3.2. Stiffness Detection

**Figure 7:**
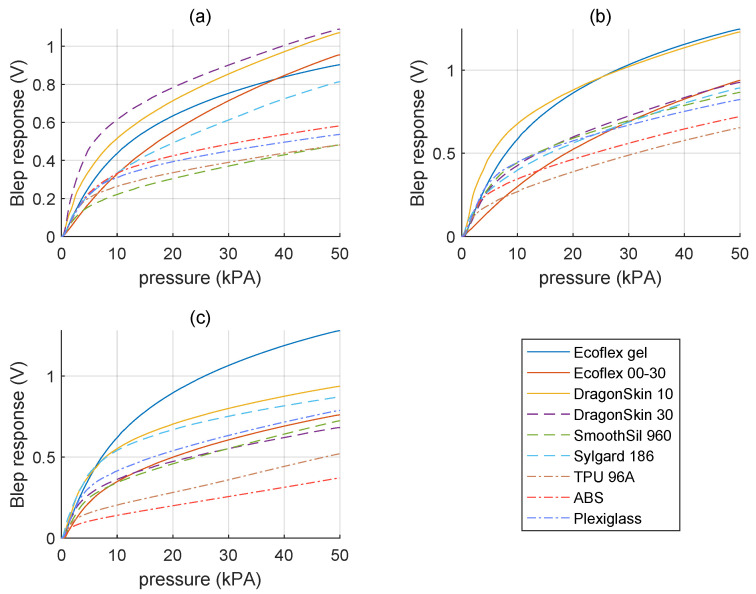
Sensor response variation to specimens with different stiffness levels during the experiment under pressure ranging from 0 to 40 kPa. Each graph corresponds to a distinct sample: (**a**) Sample 1, (**b**) Sample 2, and (**c**) Sample 3.

Figure 7 shows the indentation outcomes for the three samples, where higher voltages correspond to larger indentation. The curves reflect measurable differences in indentation depths, thus discriminating between specimens with different stiffnesses. While the theory suggests a correlation between larger and smaller indentation depths into softer and rigid materials, respectively, even minute misalignments of the two contact surfaces can introduce errors that prove wrong the previous statement.

For example, in Sample 1 (Figure 7a), Dragonskin 30 has the largest indentation. Similarly, in Sample 3 (Figure 7c), Ecoflex 30 aligns with indentation depths more indicative of soft materials, which leads to a wrong conclusion in both cases. Although these cases may be considered outliers in a controlled environment, in a real-world scenario, they are inevitable. For this reason, to increase the system’s robustness, it was preferred to include them in the dataset used in training the classifier.

The classifier underwent iterative training across all possible pressure intervals within the 0 kPa to 50 kPa range to optimize outcomes with the available data. As a result, within 70% of the evaluated intervals, the validation accuracy consistently attains values equal to or exceeding 0.9 . However, in only 30% of the intervals, the classification reaches 0.95 or higher accuracy.

**Figure 8:**
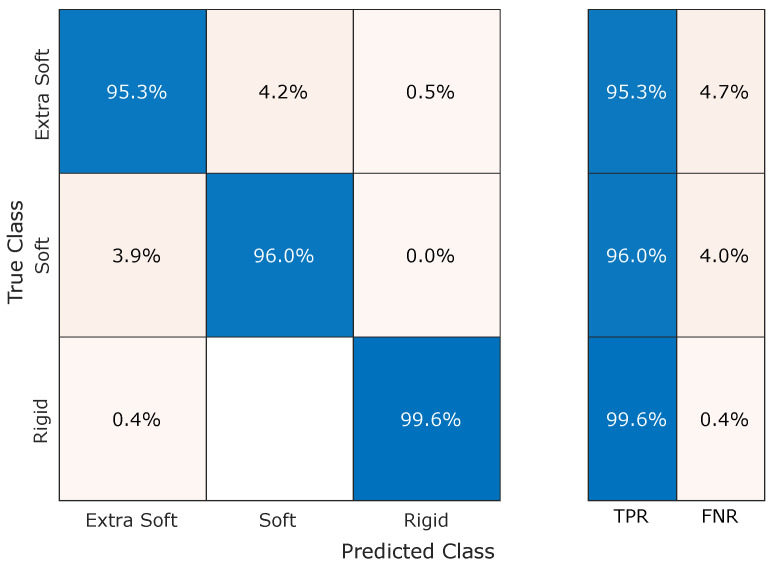
Confusion matrix of the optimized KNN results, within the optimal pressure range (10 kPa to 44 kPa). The VA result 97%, while FNR remain below 5%.

Figure 8 shows the confusion matrix for the KNN trained on the interval 10 kPa to 44 kPa. On this interval, the classification performs best, reaching a validation accuracy (VA) of 0.97 . Specifically, the false negative rate (FNR) results are always below 5% with a peak of 4.7% to extra soft material, which in 4.2% of the cases are wrongly classified as rigid. Similarly, soft materials are improperly identified as extra soft in 4% of cases, while only in 0.4% of cases rigid materials are wrongly identified as extra soft.

### 3.3. Force Detection

**Figure 9:**
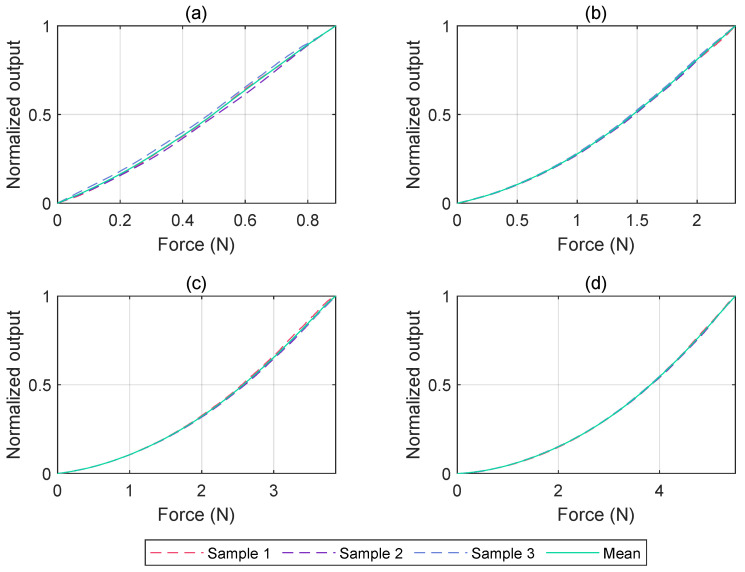
Normalized response of the three samples at different imposed pressures. For each pressure reported, the mean curve was used to generalize the approach. (**a**) 10 kPa; (**b**) 20 kPa; (**c**) 30 kPa; (**d**) 40 kPa.

The graphs presented in Figure 9 illustrate the outcomes derived from the experimental characterization of force sensing. Following theoretical expectations, the internal T-Blep pressure increases with the stiffness of the dome. Consequently, when the pressure varies from 10 kPa to 40 kPa, the detectable force range undergoes a sixfold expansion. Specifically, at 10 kPa, the maximum force recorded is 0.89 N, while at 40 kPa this value rises to 5.49 N. Similar trends are observed at intermediate pressures, particularly at 20 kPa and 30 kPa, where the maximum forces recorded are 2.31 N and 3.81 N, respectively.

Based on the explanation reported in Section 2.3.3, force reconstruction is achieved using a piecewise function. The general expression of this function can be found in (3). This methodology, coupled with real-time computation of parameters and domain intervals based on pressure, facilitates a highly precise approximation of the original data. Even in the adverse scenario tested at 40 kPa, $R^{2}$ has a value of 0.994 , approaching 1 at 10 kPa.

**Figure 10:**
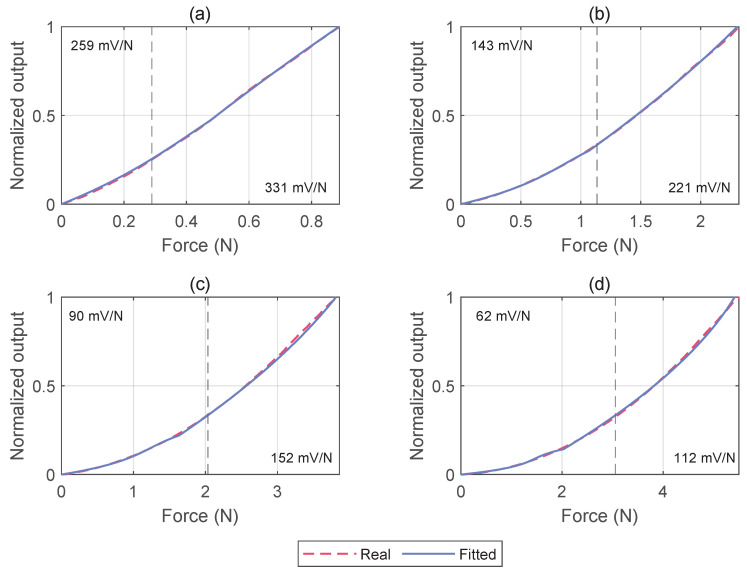
Results of force reconstruction for different pressure levels, using the piecewise function reported in (3). For each graph, the dashed line represents the domain separation point that maximizes the goodness of fit ( $R^{2}$ ). (**a**) 10 kPa; (**b**) 20 kPa; (**c**) 30 kPa; (**d**) 40 kPa.

The partitioning of the domain into two distinct parts, as outlined in Figure 10, reveals two regions characterized by different sensitivities (S) at a specified pressure. In general, an observable trend manifests, indicating a diminishing sensitivity with an increment in imposed pressure. Consistent with the previous consideration, optimal performance is achieved at 10 kPa, where the sensitivity for forces up to 0.29 N is 259 mV/N, rising to 331 mV/N for loads within the range of 0.29 N to 0.89 N. Conversely, under an imposed pressure of 40 kPa, the minimum sensitivity is 62 mV/N for forces below 3.05 N, reaching a maximum of 112 mV/N for forces within the range of 3.05 N to 5.41 N. Analogous to observations for the maximum detectable force, intermediary pressure values yield sensitivities within the range 62 mV/N to 331 mV/N, supporting the capability to modulate sensor performance through the internal pressure of the T-Blep. Table 4 provides a comprehensive overview of the sensor characteristics at 10 kPa, 20 kPa, 30 kPa and 40 kPa and the obtained results are resumed in the Supplementary Video S1 linked in the Data Availability Statement.

## 4. Conclusions

This study introduces the T-Blep, an optical soft sensor that can be tuned for a dual functionality: stiffness and force detection. Unlike conventional methods, the device integrates optical sensing elements, ensuring high resolution and robust responsiveness in the presence of electromagnetic fields. The main parts of the T-Blep are an optical sensor and an inflatable membrane, whose internal pressurization dictates the device’s functionality.

The FEA study confirms the hypothesis of using the same sensor to measure stiffness and force under various pressurization conditions. The simulation outcomes show that, within the explored pressure range, a 1 mm-thick membrane enables distinguishing between extra-soft, soft, and rigid targets, with indentations depth typically not exceeding 0.9 mm. Furthermore, the force simulation demonstrates an augmentation in the detectable maximum force as the internal pressure increases, supporting the idea of adjustable sensitivity to a broad spectrum of measurable forces.

Force detection experiments show a sixfold increase in the detectable force range as the internal T-Blep pressure varies from 10 kPa to 40 kPa. The sensor’s capability to accommodate a broad internal pressure range, spanning from 0 kPa to 40 kPa, facilitates the precise regulation of its stiffness. Consequently, this fine-tuning allows the sensor’s sensitivity to be dynamically adjusted for loads, reaching a maximum of 5.45 N. The force reconstruction method, employing a piecewise function, provides accurate approximations, even in challenging conditions, with a coefficient of determination (R2) approaching unity at 10 kPa and consistently exceeding 0.994. The sensitivity analysis highlights the ability to adjust sensor performance through internal pressure, mainly showcasing high sensitivity to loads within the range of 0–2.3 N when internal pressure results lower than 20 kPa.

Experiments focused on stiffness detection show distinguishable patterns in indentation outcomes among various samples, facilitating differentiation based on material stiffness. Nevertheless, challenges arise due to inevitable misalignments between the two contact surfaces, indicating that the results of this solution are still influenced by the load direction, a common concern in most stiffness sensors found in the literature. The optimized KNN classifier, adapted across pressure intervals, aids in mitigating this challenge, achieving a peak accuracy of 97% within the 10 kPa to 44 kPa pressure interval.

Compared to the other solution present in the literature the solution here presented does not provide any precise measurement of young modules the material taget, being the latter.

While the results of this work underscore the efficacy of this sensing approach, it must be considered that the study was conducted in a controlled environment. This setting facilitated the acquisition of a dataset with repeatable measurements but limited the exploration of potential disturbances inherent in real-world scenarios. One of the aspects that could affect the T-Blep performance is related to the possible variations of ambient light. Although subject to regular fluctuations corresponding to natural daylight changes, in the present study this aspect was not extensively assessed to guarantee insensitivity to any external light. For example, when the internal pressure of the T-Blep reaches its maximum at 40 kPa, and thus the thickness of the membrane is minimum, with the same condition, a change in the signal quality was noticed when compared to lower actuation pressure. This highlights the necessity, especially for potential integration into a robotic system, to investigate further the impact of external lights characterized by known wavelength spectra.

In contrast to existing solutions, the T-Blep presents the advantage of enabling the measurement of both force and stiffness using the same structure and transduction system. While it may not offer precise stiffness measurements like certain alternatives [10,11,12,13,14,15], the T-Blep provides a valuable classification into three categories. Although it could be seen as a limitation, it allows to directly relate sensor response to stiffness classification ignoring the force, with advantages in terms of computational efforts. Moreover, the expanded study into material recognition, as reported in the section, suggests that, with a more extensive training dataset, the same approach could offer a more precise indication of stiffness.

Concerning force detection, the T-Blep outperforms comparable solutions in measuring stiffness. With a range of 0–5.45 N, T-Blep rises the limits of 0.45 N compared to the solution of Li et al.’s 5 N while nearly tripling Weng et al.’s outputs [19,20]. The T-Blep’s sensitivity aligns with existing solutions in the same force range but can achieve up to 2.5 times higher sensitivity, surpassing the average of 121 mV/Nof the cited solution.

In conclusion, this study demonstrates how the T-Blep approach results in a versatile and adaptive sensing solution for future integration into robotic grasping systems, showcasing its potential for enabling grasping through stiffness and force detection. The study lays the groundwork for further improvements, like the invariance to misalignments and miniaturization, which is crucial for the sensor to be integrated into a robotic system.

## Figures and Tables

**Table 1:** Parameters used to model the materials in the FEA studies.

	Stiffness Study	Force Study
**Young’s modulus T-Blep,** $E_{y,b}$	1 MPa	1 MPa
**Young’s modulus Target,** $E_{y,t}$	0.1 MPa to 1 MPa	200 GPa
**Pressure, P**	0 kPa to 50 kPa	1 kPa to 40 kPa
**poisson’s ratio T-Blep,** $\nu_{b}$	0.47	0.47
**poisson’s ratio Target,** $\nu_{t}$	0.47	0.27

**Table 2:** Young’s modulus of the specimens used in the experiment, as reported in the literature.

	EcoFlex	EcoFlex	Dragon	Dragon	Sylgard	Smooth	TPU	ABS	Plexi
	Gel	00-30	Skin 10	Skin 30	186	Sil 960	95A		Glass
	0.030 [22]	0.070 [23]	0.150 [24]	0.590 [24]	1.300 [25]	1.900 [26]	67 [27]	1931 [28]	3447 [29]
		0.100 [30]	0.500 [31]	0.593 [32]	1.200 [30]	2.880 [33]			
E *		0.125 [34]	0.190 [35]		2.900 [36]	1.930 [37]			
**(MPa)**		0.068 [38]	0.151 [38]		3.00 [39]				
		0.080 [40]			1.360 [35]				
		0.069 [35]			1.58 [33]				

* E = Young’s modulus.

**Table 3:** Optimized Hyperparameters.

Number of Neighbors	Distance Metric	Distance Weight	Standardize Data
1	Mahalanobis	Inverse	no

**Table 4:** Performance of the sensor for the examined pressure.

Pressure	10 kPa	20 kPa	30 kPa	40 kPa
$R^{2}$	0.999	0.999	0.998	0.994
**Span (N)**	0–0.89	0–2.31	0–3.81	0–5.41
**FSO (V)**	0.29	0.45	0.50	0.52
**max S (mv/N)**	331	221	151	112
**min S (mv/N)**	259	143	89	62

**Figure A2:**
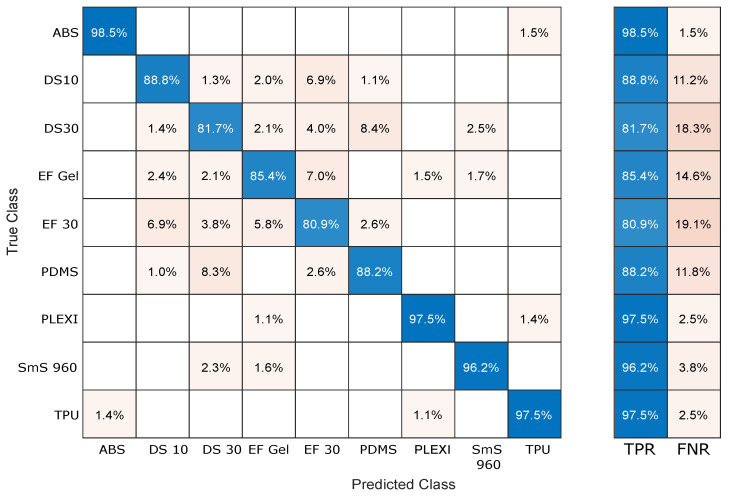
Confusion matrix of KNN trained to recognize the material.

## Data Availability

The data presented in this study are available on request from the corresponding author.

## Supplementary Materials

The following are available online at www.mdpi.com/xxx/s1, Video S1: The T-Blep: A Soft Optical Sensor for Stiffness and Contact Force Measurement.

## Abbreviations

The following abbreviations are used in this manuscript:

FEA	Finite Element Analysis
S	Sensitivity
FSO	Full scale output
$R^{2}$	coefficient of determination
KNN	k-nearest neighbors
$TiO_{2}$	Titanium dioxide particles
PDMS	Polydimethylsiloxane elastomer
ABS	Acrylonitrile butadiene styrene
VA	VAlidation Accuracy
FNR	False Negative
TPR	True Positive Rate

## Appendix A

As previously reported all the experimental activities were conducted using a custom platform. Figure A1 provides a reference to the description of the platform provided in Section 2.3.2.

## Appendix B

As reported in the Section 2.3.3, all the coefficients involved in the force reconstruction (Equation (3)) are functions of the pressure, *P*. While $f_{1}$ and $f_{2}$ could be explicitly represented by:

(A1)
$$\begin{array}{cc} f_{1}\left(\chi \right) & =a_{3}\chi^{3}+a_{2}\chi^{2}+a_{1}\chi \end{array}$$

(A2)
$$\begin{array}{cc} f_{2}\left(\chi \right) & =b_{2}\chi^{2}+b_{1}\chi +b_{0} \end{array}$$

The coefficients are obtained through the following polynomials:

(A3)
$$\begin{array}{cc} a_{3} & =0.0027\;\cdot \;P-0.1463\;\cdot \;P+2.6132\;\cdot \;P-14.0739\;\cdot \;P \end{array}$$

(A4)
$$\begin{array}{cc} a_{3} & =4.7626\times 10^{-4}\;\cdot \;P-0.1463\;\cdot \;P+2.6132\;\cdot \;P-14.0739\;\cdot \;P \end{array}$$

(A5)
$$\begin{array}{cc} a_{3} & =-2.1809\times 10^{-5}\;\cdot \;P-0.0012\;\cdot \;P+0.0367\;\cdot \;P-0.1297\;\cdot \;P \end{array}$$

(A6)
$$\begin{array}{cc} b_{3} & =-3.4233\times 10^{-5}\;\cdot \;P+0.0012\;\cdot \;P-0.0621\;\cdot \;P+0.6274\;\cdot \;P \end{array}$$

(A7)
$$\begin{array}{cc} b_{3} & =5.1183\times 10^{-5}\;\cdot \;P-0.0023\;\cdot \;P+0.1994\;\cdot \;P-1.1702\;\cdot \;P \end{array}$$

(A8)
$$\begin{array}{cc} b_{3} & =-1.5917\times 10^{-5}\;\cdot \;P+0.0015\;\cdot \;P-0.0077\;\cdot \;P+0.0951\;\cdot \;P \end{array}$$

Similarly, $x_{n}$ is associated with a polynomial of the third degree:

(A9)
$$x_{n}=-1.4071\times 10^{-5}\;\cdot \;P+0.0013\;\cdot \;P-0.0458\;\cdot \;P+0.8144\;\cdot \;P$$

The aforementioned expressions were derived via interpolating experimental data obtained under the prescribed pressure conditions.

## Appendix C

In addition to employing the 3-label KNN for differentiating extra soft, soft, or hard materials, an additional 9-label KNN was trained to identify the specific nine materials used in the experiment. Henceforth, we will refer to them as the 3-label KNN and the 9-label KNN, respectively. While the use of material labels understandably led to a reduction in overall classifier accuracy to approximately 90%, the results provided insights into which materials contributed to errors in the 3-label KNN.

The analysis revealed that, in the case of the 9-label KNN, the most significant errors occurred between materials previously classified as extra soft or soft, as reported in Figure A2. Excluding errors within the same material category, it was evident that most of the remaining errors were attributed to the misclassification of Dragonskin 30 and Ecoflex 30, accounting for 7.9%of occurrences. Similarly, PDMS and Ecoflex were confused 5.2% of the time, while confusion between Ecoflex 10 and Smooth Sil 960 occurred only 3.5% of the time.

Despite certain materials sharing characteristics, the errors in both KNNs could not be justified by similarities in Young’s modulus. As explained in the section, since stiffness detection relies on indentation depth under different pressure values, it is more plausible that an initial non-zero distance might lead to an initial false positive, which subsequent readings cannot rectify, suggesting the necessity to reduce the dependency of the sensor performance to the initial positioning.
